# Logopenic and Nonfluent Variants of Primary Progressive Aphasia Are Differentiated by Acoustic Measures of Speech Production

**DOI:** 10.1371/journal.pone.0089864

**Published:** 2014-02-28

**Authors:** Kirrie J. Ballard, Sharon Savage, Cristian E. Leyton, Adam P. Vogel, Michael Hornberger, John R. Hodges

**Affiliations:** 1 Faculty of Health Science, The University of Sydney, Sydney, New South Wales, Australia; 2 Neuroscience Research Australia, Randwick, New South Wales, Australia; 3 The University of New South Wales, Sydney, New South Wales, Australia; 4 ARC Centre of Excellence in Cognition and its Disorders, New South Wales, Australia; 5 University of Melbourne, Melbourne, Victoria, Australia; University of Barcelona, Spain

## Abstract

Differentiation of logopenic (lvPPA) and nonfluent/agrammatic (nfvPPA) variants of Primary Progressive Aphasia is important yet remains challenging since it hinges on expert based evaluation of speech and language production. In this study acoustic measures of speech in conjunction with voxel-based morphometry were used to determine the success of the measures as an adjunct to diagnosis and to explore the neural basis of apraxia of speech in nfvPPA. Forty-one patients (21 lvPPA, 20 nfvPPA) were recruited from a consecutive sample with suspected frontotemporal dementia. Patients were diagnosed using the current gold-standard of expert perceptual judgment, based on presence/absence of particular speech features during speaking tasks. Seventeen healthy age-matched adults served as controls. MRI scans were available for 11 control and 37 PPA cases; 23 of the PPA cases underwent amyloid ligand PET imaging. Measures, corresponding to perceptual features of apraxia of speech, were periods of silence during reading and relative vowel duration and intensity in polysyllable word repetition. Discriminant function analyses revealed that a measure of relative vowel duration differentiated nfvPPA cases from both control and lvPPA cases (*r* ^2^ = 0.47) with 88% agreement with expert judgment of presence of apraxia of speech in nfvPPA cases. VBM analysis showed that relative vowel duration covaried with grey matter intensity in areas critical for speech motor planning and programming: precentral gyrus, supplementary motor area and inferior frontal gyrus bilaterally, only affected in the nfvPPA group. This bilateral involvement of frontal speech networks in nfvPPA potentially affects access to compensatory mechanisms involving right hemisphere homologues. Measures of silences during reading also discriminated the PPA and control groups, but did not increase predictive accuracy. Findings suggest that a measure of relative vowel duration from of a polysyllable word repetition task may be sufficient for detecting most cases of apraxia of speech and distinguishing between nfvPPA and lvPPA.

## Introduction

**Figure 1:**
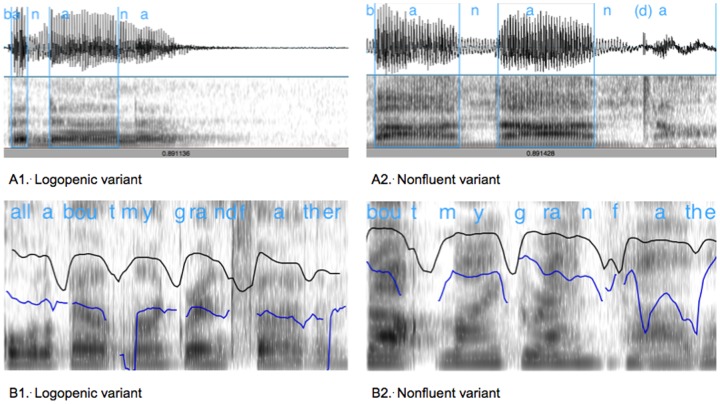
Visual examples of speech behaviors that are associated with apraxia of speech in the nonfluent variant (A2, B2) rather than the logopenic variant (A1, B1). In A1 and A2, the blue vertical lines mark the boundaries of the 2 vowels used to measure lexical stress. In B1 and B2, the black line shows vocal intensity (i.e. loudness) and the blue line fundamental frequency (i.e. vocal pitch, uncorrected) during speech; The intensity contour falls and the pitch contour breaks when the voice is silent during unvoiced sounds (e.g. ‘t’, ‘f’) or during pauses in speech. **A1:** Normal speech rate and lexical stress on polysyllabic words (here, the first ‘a’ vowel in ‘banana’ is very brief and much shorter than the second vowel) and no phonetic distortions; Total sample length 900 msec. **A2:** Slow speech rate, equal lexical stress on polysyllabic words (here, the two ‘a’ vowels in banana are of similar and longer duration, giving the perception of equal stress), and phonetic distortion on the final ‘n’ (closure of the velopharyngeal port is mistimed, blocking the nasal airflow before the tongue tip drops away from the palate, creating a sound similar to ‘d’); Total sample length 900 msec. **B1:** Normal speech rate and smooth transitions between syllables; Total sample length 1500 msec, 7 syllables. **B2:** Slowed speech rate and syllable segregation (here, the transitions between ‘-bout’ and ‘my’ and between ‘my’ and ‘grand’ are longer and more distinct in the nonfluent case), as well as distortion/substitution where the ‘f’ sound is perceived as ‘f’ but is momentarily voiced (short segment of blue line) similar to a ‘v’; Total sample length 1500 msec, 5 syllables.

One of the most challenging diagnostic problems in modern behavioural neurology and speech pathology is the differentiation of speech disorders arising from left hemisphere damage to language versus motor speech networks. In the case of Primary Progressive Aphasia (PPA), this presents a challenge in differential diagnosis of the logopenic variant (lvPPA) and the nonfluent/agrammatic variant (nfvPPA). To date, lvPPA has been associated with impaired short-term memory for sentence repetition and phonological errors of speech sound substitutions and transpositions [1]. Cases show atrophy or reduced blood flow in posterior superior and middle temporal gyri and inferior parietal lobule [2], [3]. The nfvPPA, on the other hand, has been associated with language-based grammatical errors in sentence production, along with motor speech-based effortful and halting speech production and phonetic-motoric errors of phoneme distortions [4], [5]. MR imaging studies of nfvPPA cases with motor speech impairment (i.e. apraxia of speech or AOS) have reported atrophy and hypometabolism in superior lateral premotor cortex and supplementary motor area [6] and atrophy in left posterior frontal, anterior insula, and basal ganglia regions [5], [7], [8]. An overview of the two conditions is provided in Table 1 and Figure 1.

**Table 1:** Summary characteristics of logopenic and nonfluent variants of Primary Progressive Aphasia (see text for references). Visual examples of speech behaviors associated with apraxia of speech in the nonfluent variant are provided in Figure 1.

	Logopenic variant	Nonfluent variant
Diagnostic Criteria	Phonological errors of speech sound substitutions and transpositions in naming and connected speech; Intact motor speech skills (see Figure 1: A1 and B1)	Motor speech-based impairment with slowed speech rate, phonetic-motoric distortions, syllable segregation and equalization of stress across syllables, more evident with longer stretches of speech (see Figure 1: A2 and B2); speech perceived as effortful and halting
	Impaired short-term memory for sentence repetition	Impaired sentence repetition due to motor speech deficits
	Intact grammatical sentence production	Grammatical errors in sentence production (e.g. omission of bound and free grammatical morphemes)
	Intact word comprehension and object knowledge	Intact word comprehension and object knowledge
Neurological correlates	Atrophy/reduced blood flow in posterior superior and middle temporal gyri and inferior parietal lobule	Atrophy/reduced blood flow in superior lateral premotor cortex and supplementary motor area, left posterior frontal cortex, anterior insula, and/or basal ganglia regions
Pathology	Associated with Alzheimer-type pathology	Associated with tau-positive pathology

The distinction between lvPPA and nfvPPA is relevant to clinical practice; lvPPA is associated with Alzheimer’s disease pathology [9] while nfvPPA, particularly cases with AOS, are strongly linked to tau-positive pathology [10]. Accordingly, studies using Pittsburgh compound B scans as a putative biomarker of Alzheimer’s disease have demonstrated high Pittsburgh compound B retention in virtually all cases with lvPPA and, in turn, low or normal retention in nfvPPA cases [11]. However, Pittsburgh compound B scanning is not widely available in routine practice and reliable assessment of language and speech deficits is paramount to distinguishing the two conditions.

While some studies have proposed quantitative methods for differentiating the speech production errors of lvPPA and nfvPPA, there is continued reliance on expert perceptual judgment of speech behaviours, involving listening for specific characteristics across a range of tasks [4], [5], [12], [13], [14], [15]. Ash *et al*. [13] were the first to attempt to quantify type and frequency of speech errors in nfvPPA, focusing on phonetic errors (i.e. speech sound distortions caused by motor planning impairment) versus phonemic errors (i.e. speech sound substitutions caused by representational linguistic impairment) in a story retell task. They reported 82% of errors being phonemic in nature and concluded that the speech sound errors of nfvPPA have a linguistic rather than motoric basis. However, this method of speech analysis still relies on perceptual judgments of speech.

The challenge of reliably differentiating motor and linguistically-based speech errors in PPA has led some to propose that nfvPPA and lvPPA may not be distinct entities [16]. Leyton *et al*. [15], by contrast, reported that classification of the variants, based on a combination of traditional neuropsychological tests and perceptual judgment on presence or absence of specific speech behaviours [1], showed a strong correlation with results of Pittsburgh compound B scanning; 12 of 13 individuals diagnosed with lvPPA demonstrated increased Pittsburgh compound B uptake consistent with diffuse Alzheimer-type pathology while only two of eight individuals with nfvPPA were Pittsburgh compound B positive. Despite this, perceptual approaches to diagnosis are highly susceptible to clinician expertise and perceptual bias [17], [18] motivating development of reliable objective metrics of speech behaviours.

In the case of nfvPPA, it is estimated that more than 80% of individuals may demonstrate the speech motor planning/programming impairment of AOS [19], and in many cases, it is the presenting symptom [20]. AOS is not associated with lvPPA [21] and thus the ability to detect AOS could provide a good discriminator. Currently, expert perceptual judgment of speech errors is the gold standard for clinical diagnosis of AOS [1], [4], [5] as objective acoustic measurements of speech production for reliably detecting the motor-based errors of AOS in this group have not been identified.

Duffy [4] reported the four most common perceptual features of AOS observed in nfvPPA are slowed speech rate, phoneme distortions, syllable segregation and equalization of lexical stress across syllables within words, each occurring in ≥75% of the patients tested. Syllable segregation occurs when pauses or hesitations are inserted between syllables and words [22] (see Table 1 and Figure 1). Equalization of lexical stress occurs when duration or loudness of de-stressed syllables in words (i.e. ‘no’ in dinosaur or ‘po’ in potato) is increased relative to stressed syllables [23], [24], [25], due presumably to impairment of fine rapid control of laryngeal and supra-laryngeal movements to produce these subtle differences. Both pausing and lexical stress changes can indicate difficulty with planning spatial, temporal and amplitude parameters of multi-articulator movements for speech and result in slowed speech rate. Duffy’s [4] observations in PPA are highly consistent with studies examining acquired AOS subsequent to stroke [22]. Here, we focus on syllable segregation and lexical stress, as measurement from digital recordings of speech is straightforward, does not rely on expert judgments, and can be implemented in automated software routines [24], [25], [26], [27], [28], which standardize and streamline data collection and analysis.

The aim of this study was firstly to evaluate a set of reliable instrumental acoustic measures for differentiating nfvPPA and lvPPA focusing on (a) syllable segregation, captured by measurement of periods of silence in connected speech, and (b) altered stress patterns within words, captured by measurement of relative duration and loudness of syllabic vowels within words.

We hypothesized the following:

1. In a paragraph reading task, proportion of silence time will be higher, median silence duration longer, and variability of silence duration higher in nfvPPA than in lvPPA.
2. Duration and loudness of vowels will be more equal across syllables within polysyllabic words in nfvPPA than in lvPPA.
3. Individuals with nfvPPA will perform differently on measures of silence and lexical stress compared to a healthy age-matched control group due to their speech motor impairment, but lvPPA will perform similarly to control participants.
4. Measures of syllable segregation and/or altered lexical stress should correlate significantly with patterns of atrophy seen in nfvPPA, such as premotor cortex and supplementary motor area, as well as inferior frontal and anterior insula regions [5] [10], [29], [30].

A secondary aim was to use those measures most sensitive to distinguishing lvPPA and nfvPPA to create a diagnostic algorithm for the sample.

## Method

### Ethics Statement

Written informed consent was obtained from the participant. When the participant did not fully comprehend the consent process and study requirements, as judged by the participant themselves or the neurologist or neuropsychologist, a legally authorized primary caregiver provided written informed consent. All procedures conformed to the Declaration of Helsinki (BMJ 1991; 302: 1194) and were approved by the South Eastern Sydney and Illawarra Area Health Service and the University of New South Wales human ethics committees.

### Participants

Forty-one individuals (23 females and 18 males; mean = 68.3 years, standard deviation = 8.3, range 50 to 83), from a consecutive sample of patients evaluated between January 2009 and June 2012 at the specialist Frontotemporal Dementia Research Group at Neuroscience Research Australia, met the inclusion criteria: (a) an initial diagnosis of lvPPA or nfvPPA [1], representing consensus across two neurologists experienced in PPA, (b) no diagnosis of other neurologic conditions such as motor neuron disease, corticobasal degeneration, progressive supranuclear palsy, stroke or traumatic brain injury at time of study, (c) fluent speakers of English prior to onset of PPA (non-native English speakers were not excluded; see Table 2), and (d) a high quality audio recording of a reading of the Grandfather Passage [31] and the polysyllable word repetition subtest of the Sydney Language Battery (SYDBAT) [32].

**Table 2:** Demographic and formal testing data presented for logopenic and nonfluent variant Primary Progressive Aphasia groups and healthy controls with statistical comparisons between the logopenic and nonfluent variant groups for continuous variable reported.

Variable/Test	Logopenic variant	Nonfluent variant	Healthy control
Sample size	21	20	17
Age	67.2 (7.1)	69.5 (9.4) ^ns^	68.6 (7.4)
Sex	5 Male	13 Male	7 Male
Education	12.4 (3.6)	13.1 (3.2) ^ns^	14.2 (3.5)
Estimated Time Post-Onset (yrs)	4.2 (2.8)	2.9 (1.6) ^ns^	
English First Language^1^	20	13	17
Right-Handed	19	20	16
Normal/corrected hearing and vision^2^	18	17	17
Pittsburgh compound B positive^3^	13/13	3/10	
Apraxia of Speech^4^	0/21	14/20	0/17
FTLD-mCDR Scale ^5^	5.44 (3.9)	5.55 (4.2) ^ns^	0.3 (0.5)
Mini-Mental State Examination (/30) ^6^	21.9 (4.5)	24.8 (4.8) ^ns^	29.5 (0.9)
SYDBAT Naming (/30)	13.0 (6.5)	21.5 (6.2) ***	
SYDBAT Semantic (/30)	25.5 (3.1)	24.9 (5.3) ^ns^	
SYDBAT Comprehension (/30)	26.2 (2.2)	27.5 (3.0) ^ns^	
SYDBAT Repetition (/30)	24.1 (6.9)	25.4 (5.3) ^ns^	
Test of Reception of Grammar (/80)	55.0 (19.0)	65.0 (12.6) ^ns^	

Ns = nonsignificant; *** p<0.001; **^1^** All fluent English speakers premorbidly, by self or family report; **^2^** 3 logopenic and 3 nonfluent participants reported cataracts, one reported a macular tear; polysyllable words were repeated after the examiner and all patients were able to read the Grandfather Passage (large font) unassisted; the number of affected cases is balanced across patient groups; ^3^ Pittsburgh compound B positron emission tomography scanning using cut-off of 1.5 for neocortical standardized uptake value ratios; ^4^ Cases of nonfluent PPA without clinical diagnosis of AOS are included, as acoustic measures may be more sensitive than perceptual judgments to motor speech behaviors [38]; ^5^ Frontotemporal Lobar Degeneration-modified Clinical Dementia Rating Scale with score >3 indicating impairment [45], data available for 12 controls; ^6^ Lower limit of normal performance is 28/30 for 60–69 year olds and 27/30 for 70–79 year olds [69], data available for 12 controls.

Of the 41 participants, 21 were diagnosed with lvPPA and 20 were diagnosed with nfvPPA, following extensive neurological and neuropsychological evaluation as part of a larger program of research described elsewhere [15]. Demographic information and performance on neuropsychological tests is reported in Table 2.

Twenty-three of the 41 PPA participants (13 lvPPA, 10 nfvPPA) underwent a Pittsburgh compound B scan at the Austin Hospital, Melbourne, following our previously published protocol [15]. Standardised Uptake Values for Pittsburgh compound B were calculated for all brain regions examined and Standardised Uptake Value ratios were generated by dividing all regional values by the cerebellar cortex values. The cut-off for high and low neocortical Pittsburgh compound B retention was set at Standardised Uptake Value ratio of 1.5 [33], [34]. Consistent with previous studies [20], [15], all 13 lvPPA cases tested positive for Pittsburgh compound B, while only 3 of the nfvPPA cases tested positive.

Seventeen healthy age- and education-matched controls (10 females and 7 males; mean = 68.6 years, standard deviation = 7.4, Range 50 to 79) were recruited for comparison. All were native English speakers with no reported history of speech, language, or neurological disorder.

### Procedures and Equipment

Two tasks were selected for acoustic analysis:

(a) a reading of the Grandfather Passage, recorded with a Marantz PMD671 solid state recorder and AudioTechnica ATM75 cardioid headset microphone positioned 10 cm from the mouth, with 48 kHz sampling frequency and 16-bit quantization in.wav format., and

(b) the 30-item SYDBAT polysyllable word repetition subtest [32], recorded with a range of digital audio and video-recording devices with the microphone placed at a constant mouth-to-microphone distance.

Both samples were obtained within one month of each other, and were analysed using Praat speech analysis software (version 5.0.56 [35]) to display and analyse the acoustic waveform and spectrogram (i.e. frequency and amplitude over time) of the speech samples. While duration, intensity, and fundamental frequency of polysyllable words can be measured; the latter is not informative for examining lexical stress in this task [36], [37], [38] and was not used here.

### Dependent Measures

#### Syllable Segregation

The reading samples were used to examine syllable segregation by studying the features of hesitations and within and between word silences using three measures [39], [40]:

1. proportion of silence time (summed duration of silences/total duration of speech sample),
2. median duration of silences, and
3. variability of silence duration (median absolute deviation of silence duration).

First, the intensity contour across the speech sample was generated using the generic algorithm in Praat. Periods of silence were then defined using three criteria: (a) windows where the intensity contour fell below the threshold of 0.65 of the distance between the minimum intensity and the reference intensity (i.e. 0.95 of the maximum intensity, considered more robust to irregular bursts of energy than maximum, medial or modal intensities [39], (b) minimum duration of 15 ms, and (c) minimum intervening speech duration of 30 ms (for detailed method, see Method S1). Silences at the start and end of each sample and bursts of intensity due to nonspeech behaviour (e.g. laughing, coughing) were removed prior to measurement.

#### Lexical Stress

The polysyllable word samples were used to examine lexical stress. Lexical stress can be marked by variations in duration and intensity, related to features of slow rate (i.e. extended phoneme durations) and excess and equal stress within words. Ten words were selected from the SYDBAT word repetition subtest, five with a strong-weak stress pattern over the first two syllables (i.e. butterfly, bicycle, caterpillar, dinosaur, stethoscope) and five with a weak-strong pattern (i.e. banana, computer, pagoda, potato, thermometer). These words are ideal for acoustic measurement of vowels: the vowels are adjacent to plosive (e.g. p, t), fricative (f, s, th), and nasal (m, n) phonemes that have clear boundaries in the waveform and/or spectrogram visually displayed within Praat, allowing reliable identification of vowel onset and offset for measuring duration. Note that the post-vocalic/pre-consonantal ‘r’ in butterfly, caterpillar, and thermometer is not realised in Australian-English.

For each word, Pairwise Variability Indices (PVI) were calculated from measurement of vowel duration (ms) and peak intensity of the vowel (dBSPL) from the first two syllables of the word, using the formula below and converted to absolute values [23], [25], [27]. 
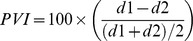
where d is duration in ms or intensity in dBSPL of the first (1) and second (2) vowel.

PVI represents the normalized magnitude of difference in vowel duration or intensity for a word, allowing for comparison across different speaking rates and vocal loudness levels, respectively. Values closer to zero reflect more equal stress (i.e. abnormal stress for any of the selected words). For each individual, four median PVI values were analysed, representing the relative duration and intensity of vowels in strong-weak and weak-strong words (PVI_Duration_SW, PVI_Duration_WS, PVI_Intensity_SW, and PVI_Intensity_WS).

### Statistical Analyses

All measures were screened for skewness and kurtosis in SPSS (IBM, Mac Version 20). Only median duration of silences and variability of silence duration, and their transformed distributions, were significantly non-normal and so nonparametric tests were used for those univariate analyses. Proportion of silence time values were arcsin transformed (arcsinPPT).

Initially, univariate ANOVA, with Tukey-HSD/Kramer post-hoc testing, or the Independent Samples test of medians were conducted to test for a main effect of group (lvPPA, nfvPPA, Control) on each dependent measure. As the purpose was to explore potential predictors to enter into the multivariate discriminant function analyses (DFA), alpha level was held at 0.05. The DFA, implemented in the Linear Regression function of SPSS, was then run on data for the lvPPA and nfvPPA groups to identify any combination of variables that significantly predicted assignment to patient group. In preparation for the DFA, a correlation matrix was generated for all dependent measures of speech returning significant effects in univariate ANOVAs, to identify and exclude cases of multicollinearity (r>0.80, [41], [42]). Mahalanobis distance was calculated to check for multivariate outliers [42]. Based on the recommendation of 10 participants per variable and a total sample size of 41 patients [41], a maximum of four variables were entered into the model for each DFA.

### Image acquisition and voxel-based morphometry

Voxel-based morphometry was used to identify grey matter intensity changes across groups on a voxel-by-voxel basis using structural MRI data. Thirty-seven of the 41 PPA participants (18 lvPPA and 19 nfvPPA), as well as 11 of the 17 healthy controls, underwent whole-brain T1-weighted imaging using a 3T Philips MRI scanner with standard quadrature head coil (8 channels), using the following sequences: coronal orientation, matrix 256×256, 200 slices, 1×1 mm^2^ in-plane resolution, slice thickness 1 mm, echo time/repetition time = 2.6/5.8 ms, flip angle α = 19°.

MRI data were analysed with FSL-VBM from the FMRIB software package (http://www.fmrib.ox.ac.uk/fsl/fslvbm/index.html). Briefly, structural images were extracted using the brain extraction tool (BET). Tissue segmentation was then carried out on these images using FMRIB’s Automatic Segmentation Tool (FAST). The resulting grey matter partial volumes were then aligned to the Montreal Neurological Institute standard space (MNI152) using the FMRIB non-linear registration approach (FNIRT), which uses a b-spline representation of the registration warp field. A study-specific template was created, combining lvPPA, nfvPPA and healthy control images, to which the native grey matter images were re-registered nonlinearly. The registered partial volume maps were then modulated by dividing by the Jacobian of the warp field. This step was carried out to correct for local expansion or contraction. The modulated segmented images were then smoothed with an isotropic Gaussian kernel with a sigma of 3 mm.

A voxel-wise general linear model was applied to investigate grey matter intensity differences via permutation-based non-parametric testing with 5000 permutations per contrast. In a first step, differences in cortical grey matter intensities between patients (lvPPA, nfvPPA) and control participants were assessed, contrasting lvPPA vs. control participants, nfvPPA vs. control participants, lvPPA vs. nfvPPA and nfvPPA vs. lvPPA (*P*<0.05 FDR corrected, clusters overlaid on the MNI standard brain and reported at t>3.27; see Figure S1). Next, correlations between the speech measures emerging as strongest predictors of diagnostic group in the multivariate DFA analyses and regions of reduced grey matter intensity were investigated in the patient groups combined with healthy controls. This procedure serves to increase the statistical power to detect brain-behaviour relationships across the entire brain by achieving greater variance in behavioural scores [43], [44]. For this analysis, a new study-specific template was generated dichotomising the samples as PPA (pooled lvPPA and nfvPPA) or control and diagnostic category was entered as a nuisance variable in the covariate analysis. For statistical power, a covariate only statistical model with a [–1] t-contrast was used, providing an index of association between grey matter intensity and speech production. A region of interest (ROI) mask was created for the following brain areas (bilateral inferior and middle frontal regions, precentral gyrus, and supplementary motor areas), which were identified in previous independent studies as being related to AOS (see Introduction). Anatomical locations of significant results were overlaid on the MNI standard brain, with maximum coordinates provided in MNI stereotaxic space. Anatomical labels were determined with reference to the Harvard-Oxford probabilistic cortical atlas. The inter-patient comparisons and the covariate analyses are reported at *P*<0.05 false discovery rate (FDR) corrected for multiple comparisons over significant clusters.

## Results

### Participant Demographic and Test Performance

The lvPPA and nfvPPA groups were not different in mean age or education (p>0.05) (see Table 2). Average disease severity scores, as measured by the Frontotemporal Lobar Degeneration-modified Clinical Dementia Rating Scale (FTLD-CDR) [45] and Mini-Mental State Examination (MMSE) [46], for both groups fell in the mild range. The lvPPA group performed more poorly than the nfvPPA group on the SYDBAT naming subtests, consistent with a primarily lexically-based impairment. Of the nfvPPA group, 14 (70%) received a consensus diagnosis of AOS by expert clinicians based on presence/absence of descriptive features during neuropsychological testing (e.g. effortful and halting speech and distorted phonemes); no lvPPA cases were judged to have AOS. Thirteen nfvPPA cases demonstrated grammatical errors, ranging from minimal to moderate severity, during conversation and picture description tasks.

### Speech Analyses

#### Univariate Analyses

Results of the omnibus ANOVAs, relevant post-hoc comparisons, and group means with standard deviations for each dependent measure of speech are presented in Table 3. As expected, significant group effects were found for all three measures of silence during reading, as well as for the PVI_Duration measure for both WS and SW words from the repetition task. Post-hoc tests for silence behaviours showed elevated median silence duration and variability of silence duration for nfvPPA patients when compared both with controls (*P*<0.001), and with the lvPPA groups (*P*<0.05). Similarly, post-hoc tests for PVI_Duration revealed that the nfvPPA group had smaller PVI values (i.e. perceived as more equal stress) for both weak-strong and strong-weak words, compared to controls (*P*<0.05) and lvPPA (*P*<0.001). Contrary to expectation, however, lvPPA patients showed an equivalent impairment in the proportion of silence time as nfvPPA patients, and when compared to controls were also significantly elevated in their silence duration and variability (*P*<0.05), although not to the same degree as nfvPPA participants. PVI_Duration, however, was equivalent between lvPPA and controls. PVI_Intensity did not differentiate the three groups.

**Table 3:** Comparison between healthy controls and individuals with logopenic (lvPPA) or nonfluent variant (nfvPPA) Primary Progressive Aphasia on acoustic measures of speech.

Measure	Omnibus test^1^	Group	Mean (SD)	Post-hoc tests
Proportion Silence Time 1	*F*(2,55) = 6.062, *P* = 0.004	lvPPA	0.61 (0.13)	lvPPA - nfvPPA ^ns^
		nfvPPA	0.62 (0.17)	lvPPA – Control *
		Control	0.48 (0.09)	nfvPPA-Control **
Median Silence Duration (ms) ^2^	*P* = 0.000	lvPPA	125.9 (50.0)	lvPPA-nfvPPA *
		nfvPPA	236.1 (208.8)	lvPPA-Control *
		Control	83.3 (27.2)	nfvPPA-Control ***
Variability of Silence Duration ^2^	*P* = 0.018	lvPPA	86.5 (47.7)	lvPPA-nfvPPA *
		nfvPPA	158.6 (125.9)	lvPPA-Control *
		Control	49.7 (27.6)	nfvPPA-Control *
Median PVI_Duration_WS	*F*(2,55) = 9.653, *P* = 0.000	lvPPA	–116.2 (18.3)	lvPPA-nfvPPA ***
		nfvPPA	–87.4 (26.9)	lvPPA-Control ^ns^
		Control	–109.7 (18.9)	nfvPPA-Control **
Median PVI_Duration_SW	*F*(2,55) = 8.326, *P* = 0.001	lvPPA	89.6 (17.0)	lvPPA-nfvPPA ***
		nfvPPA	63.9 (26.2)	lvPPA-Control ^ns^
		Control	80.7 (16.0)	nfvPPA-Control *
Median PVI_Intensity_WS	*F*(2,55) = 0.728, *P* = 0.487			
Median PVI_Intensity_SW	*F*(2,55) = 0.593, *P* = 0.446			

1 Arcsin transformed; ^2^Median Silence Duration and Variability of Silence Duration tested with Independent Samples Median Test; ns = nonsignificant, *P<0.05, ** *P*<0.01, *** *P*<0.001. PVI = Pairwise Variability Index, WS = weak-strong words (e.g. potato), SW = strong-weak words (e.g. dinosaur).

#### Multivariate Discriminant Function Analysis (DFA)

Testing for multicollinarity, bivariate correlation analyses revealed high correlations between median silence duration and variability of silence duration (r>0.80). As initial DFA analyses showed that median silence duration yielded multivariate outliers, variability of silence duration was used. PVI_duration_WS and PVI_Duration_SW were not highly correlated with each other. Therefore, variability of silence duration, proportion of silence time (arcsinPST), PVI_duration_WS and PVI_Duration_SW were entered as predictors, with PPA variant (lvPPA, nfvPPA) as the dependent variable. In this model, Mahalanobis distance did not exceed the critical χ^2^ value of 18.47 for any cases (*df = *4; α = 0.001), indicating multivariate outliers were not a concern. The model accounted for 49.0% of the variance for the sample. Removing the nonsignificant predictor PVI_duration_SW resulted in a nonsignificant drop to 46.1%. The resulting Model 1 with three predictors is presented in Table 4.

**Table 4:** Results of multivariate discriminant function analyses with aphasia variant as the dependent variable.

		Unstandardized Coefficient		Standardized Coefficient		
Predictor Variables	*r* ^2^	B	Standard Error	Beta	*t*	*P*
**Model 1**						
*F*(3,37) = 10.541, *P* = 0.000	0.461					
(Constant)		2.387	0.460		5.192	0.000
Proportion of silence time ^1^		–2.057	0.638	–0.611	–3.224	0.003
Variability of silence duration		2.991	0.987	0.591	3.030	0.004
Median PVI_Duration_WS		0.010	0.003	–0.520	–3.785	0.001
						
**Model 2**						
*F*(2,35) = 15.471, *P* = 0.000	0.471					
(Constant)		1.763	0.247		7.129	0.000
Median PVI_Duration_WS		–0.008	0.003	–0.422	–2.771	0.009
Median PVI_Duration_SW		–0.007	0.003	–0.347	–2.278	0.029

Model 1 includes all participants; Model 2 excludes three nfvPPA patients with contradictory findings on Pittsburgh compound B scanning; ^1^ arcsin transformed.

**Figure 2:**
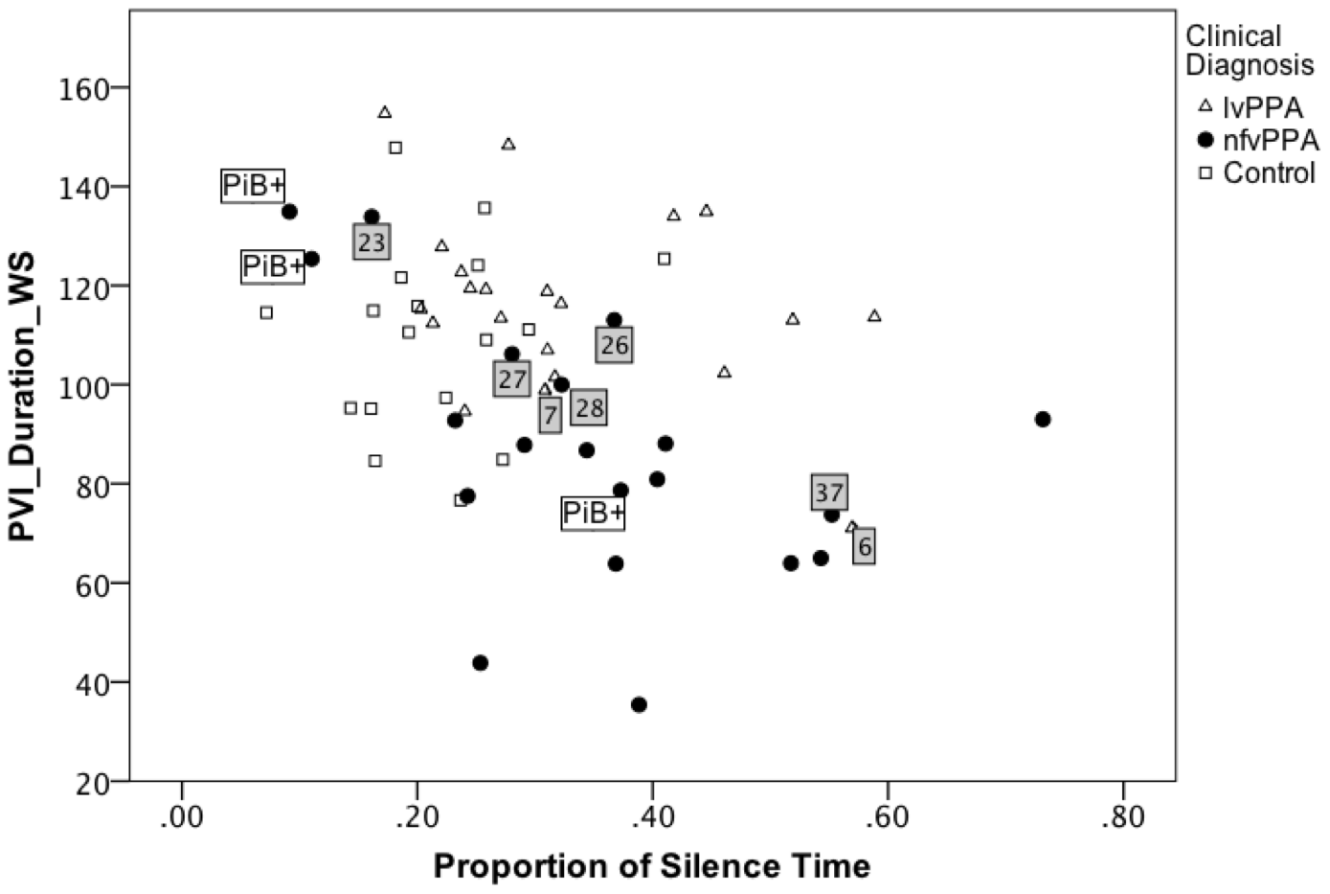
Pairwise variability of vowel duration as a function of proportion of silence time. The relationship between median Pairwise Variability Index for vowel duration in weak-strong polysyllabic words (PVI_Duration_WS) and proportion of silence time in reading is shown for patients with logopenic variant (lvPPA) and nonfluent variant (nfvPPA) Primary Progressive Aphasia and healthy age-matched adults. For PVI_Duration_WS, smaller values represent more equal stress across the first two syllables of words. Grey squares mark the 7 of 41 patient cases misidentified by the discriminant function Model 1 (lvPPA cases 6 and 7, nfvPPA cases 23, 26, 27, 28, and 37). PiB+ marks the three nfvPPA patients with contradictory positive findings on Pittsburgh compound B scanning, indicative of Alzheimer pathology and more commonly associated with the logopenic variant. Control participants were not included in the discriminant function analysis but are shown here for comparison.

**Figure 3:**
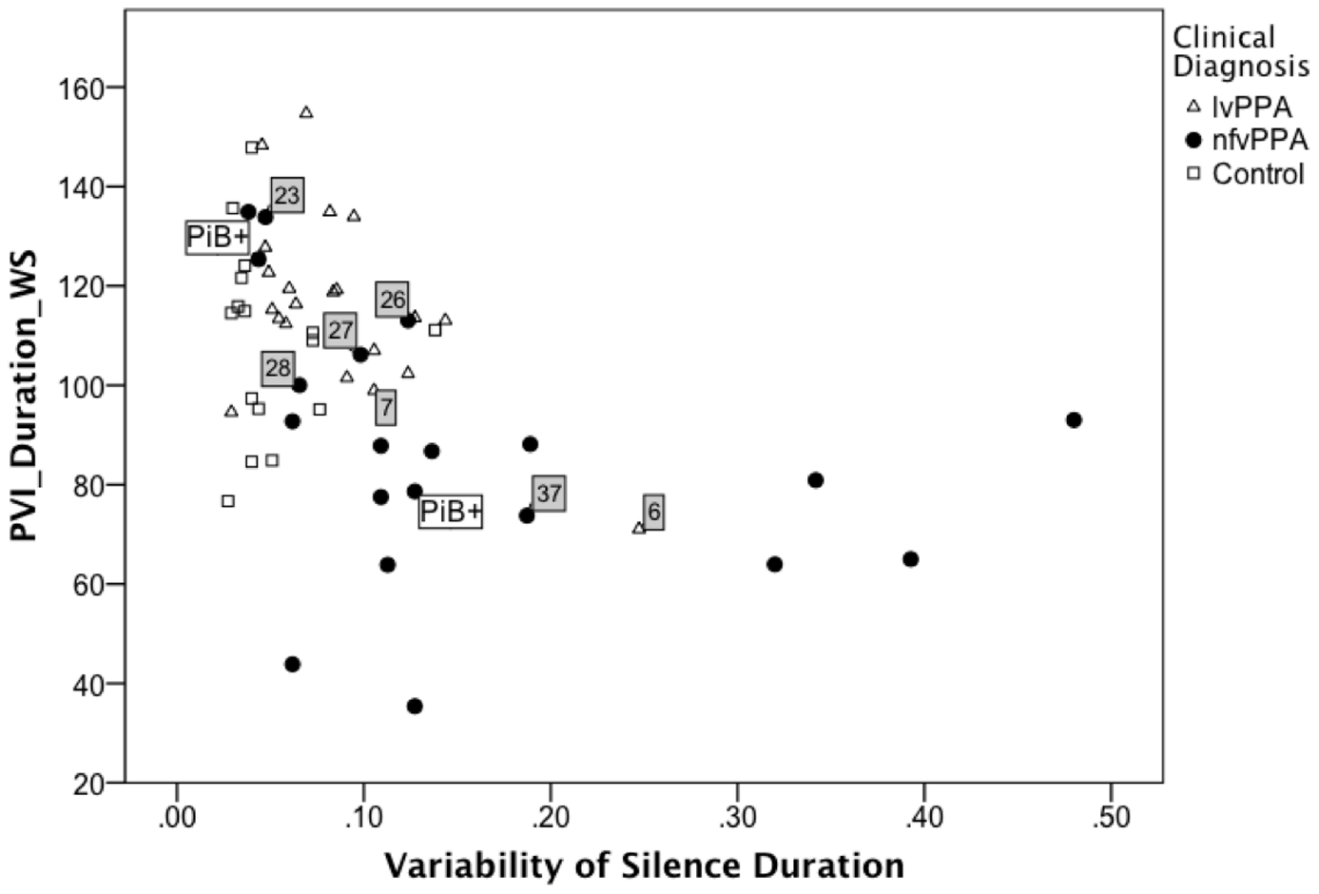
Pairwise variability of vowel duration as a function of variability of silence duration. The relationship between median Pairwise Variability Index for vowel duration in weak-strong polysyllabic words (PVI_Duration_WS) and variability of silence duration in reading is shown for patients with logopenic variant (lvPPA) and nonfluent variant (nfvPPA) Primary Progressive Aphasia and healthy age-matched adults. For PVI_Duration_WS, smaller values represent more equal stress across the first two syllables of words. Grey squares mark the 7 of 41 patient cases misidentified by the discriminant function Model 1 (lvPPA cases 6 and 7, nfvPPA cases 23, 26, 27, 28, and 37). PiB+ marks the three nfvPPA patients with contradictory positive findings on Pittsburgh compound B scanning, indicative of Alzheimer pathology and more commonly associated with the logopenic variant. Control participants were not included in the discriminant function analysis but are shown here for comparison.

**Figure 4:**
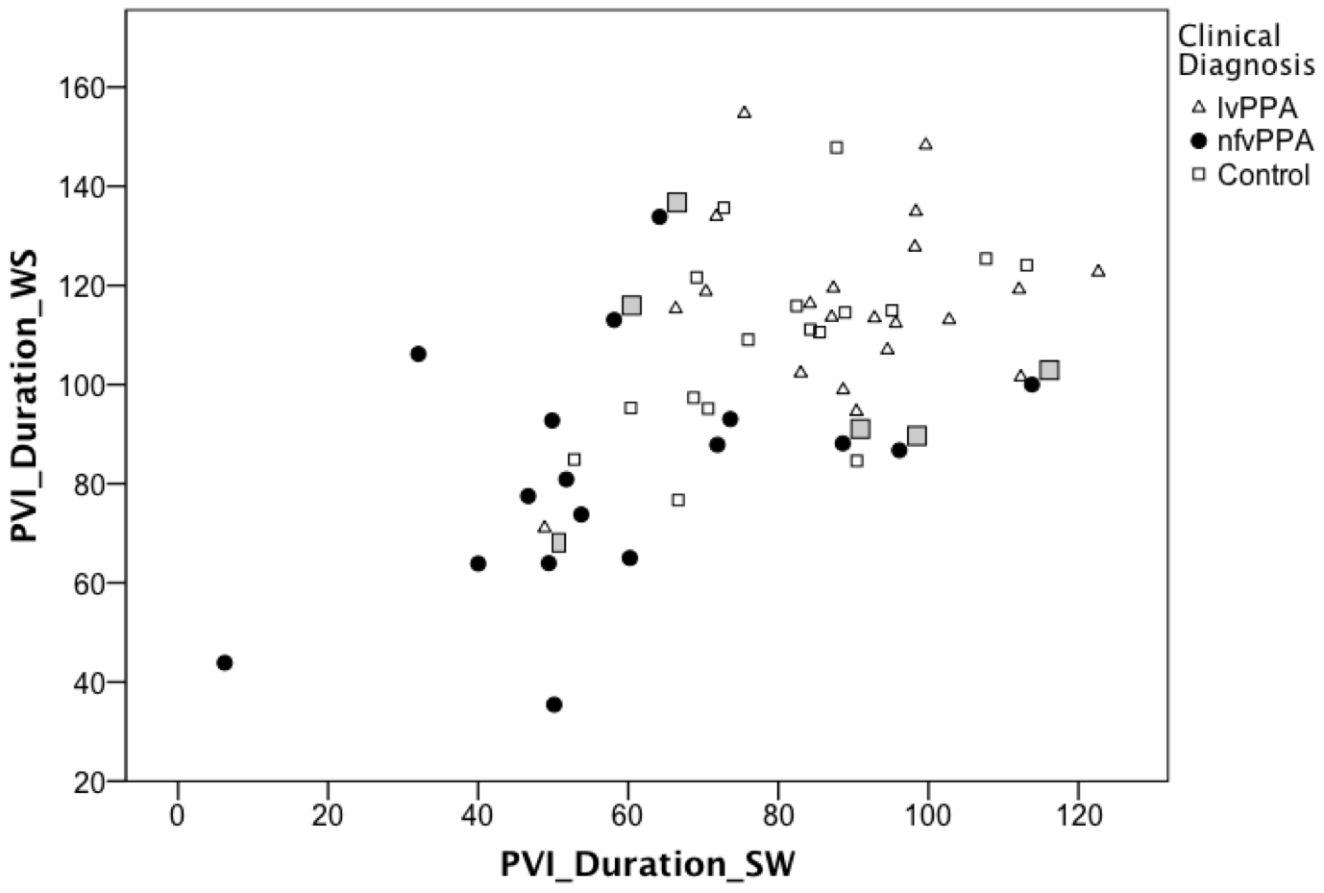
Pairwise Variability of vowel duration in weak-strong versus strong-weak polsyllable words. The relationship between median Pairwise Variability Index for vowel duration in weak-strong and strong-weak polysyllabic words (PVI_Duration_WS, PVI_Duration_SW, respectively) for individuals with logopenic variant (lvPPA) and nonfluent variant (nfvPPA) Primary Progressive Aphasia and healthy age-matched adults. Smaller values represent more equal stress (i.e. more similar duration) across the first two syllables of words. Consistent with this, individuals with PVI_Duration_WS less than about 110 *and* PVI_Duration_SW less than about 80 are confidently diagnosed as nfvPPA by the model. Grey boxes indicate 6 patient cases misclassified in a discriminant function analysis (lvPPA case 6, nfvPPA cases 23, 26, 28 labeled in previous figures, and nfvPPA cases 35 and 41 being the two cases with PVI_Duration_SW values between 80 and 100). The three nfvPPA patients with contradictory positive findings on Pittsburgh compound B scanning were excluded from the analysis and are not shown here. Control participants were not included in the discriminant function analysis but are shown here for comparison.

To examine the data at an individual case level, median PVI_Duration_WS was plotted against proportion of silence time (see Figure 2) and variability of silence duration (see Figure 3). Three nfvPPA cases were noted to have uncharacteristically high values for PVI_Duration_WS and low values for the two silence measures. Two of these nfvPPA cases had positive Pittsburgh compound B scans, indicating the presence of Alzheimer pathology. To explore the possibility that these nfvPPA patients with positive Pittsburgh compound B findings might be behaving differently from nfvPPA with negative findings and overly influencing the DFA model, data from all three positive cases were removed and the DFA re-run. All three were removed to minimize bias. The resulting model is reported in Table 4 (Model 2), where PVI_Duration_WS and PVI_Duration_SW emerged as the significant predictors of diagnostic group accounting for 47.1% of variance in the sample (see Figure 4). Proportion of silence time and variability of silence duration did not contribute significant changes to the model.

To ensure that data from the 1 lvPPA and 7 nfvPPA cases, who were non-native English speakers, were not overly influencing the modeling, the univariate and multivariate analyses were rerun with these cases excluded (see Tables S1 and S2, respectively). The PVI_Duration measures survived as variables that significantly differentiated the lvPPA and nfvPPA groups, while no silence measures survived.

#### Diagnostic accuracy

To examine the diagnostic accuracy of the two models, the unstandardised B coefficients from each Model (see Table 4) were used as weights on the significant dependent measures. For any given patient in this sample, the following formula can be used to compare the model-based diagnosis to the clinical diagnosis (example given for Model 2); the formula returns a continuous index incorporating all significant predictors (a Silence+Duration Index for Model 1, a Duration Index for Model 2) or, by rounding to zero decimal places, a value of 0 for lvPPA or 1 for nfvPPA.
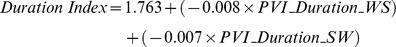



Applying the formula from Model 1, 34 of the 41 patients (83%, 19/21 lvPPA and 15/20 nfvPPA) were assigned to the expected diagnostic group. For the nfvPPA group, 14 of 20 had been clinically judged to have some degree of AOS, the remainder judged to have only aphasia. Agreement between the model-based diagnosis and the expert diagnosis for presence of AOS was 75% (15/20). The nfvPPA cases misclassified as lvPPA (cases 23, 26, 27, 28, and 37; see Figures 2 and 3) tended to have PVI_Duration_WS values ≥100, proportion silence time <0.39, and variability of silence duration value <0.12, consistent with more fluent speech with a normal rhythm. Misclassified lvPPA cases (cases 6 and 7 in Figures 2 and 3) tended to have a PVI_Duration_WS value <100, proportion silence time >.30, and variability of silence duration value >0.10, consistent with less fluent and more equally stressed speech.

Applying the formula from Model 2, 32 of the 38 patients (84%, 20/21 lvPPA and 12/17 nfvPPA) were assigned to the expected diagnostic group. Of the 5 nfvPPA cases misclassified as lvPPA (cases 23, 26, 28, 35, and 41; see Figure 4), all had PVI_Duration for only one of the stress types within the nfvPPA range and only one of the five cases was judged *a priori* to have AOS. For the nfvPPA group, 12 of 17 had been clinically judged to have some degree of AOS (4/12 with AOS alone, 5/12 with some degree of concomitant dysarthria and agrammatism and 3/12 agrammatism but no dysarthria) [1]. Of the other 5/17 cases, three were judged to have dysarthria but no agrammatism and two to have agrammatism alone. Agreement between the model-based diagnosis and the expert diagnosis for presence of AOS was 88% (15/17; 16/20 for the whole sample); in one disagreement case, the model identified AOS and the expert judgment indicated mild dysarthria alone and in the other case the model identified no AOS and the expert judgment indicated AOS, mild dysathria, and minimal agrammatism. Cases that satisfied both conditions of PVI_Duration_WS value below ∼110 and PVI_Duration_SW value below ∼80 were confidently diagnosed as nfvPPA by Model 2 (see Figure 4). The one misdiagnosed lvPPA participant (case 6) had low PVI-duration values for both WS and SW words, as well as high values for median silence duration and variability of silence duration within the lvPPA group (Figures 2 and 3), consistent with the nfvPPA pattern.

### Brain-Behaviour Correlations

Group comparisons between patients and controls (see Figure S1) showed that the lvPPA group had a region of extensive left-sided cortical atrophy encompassing superior, middle and inferior temporal gyri, extending to the temporo-parieto-occipital junction as well and anteriorly into the inferior frontal gyrus, pars opercularis, and insula cortex. By contrast, the nfvPPA group demonstrated bilateral atrophy, which was much more circumscribed and involved the inferior frontal gyrus, pars opercularis, and middle frontal gyrus, as well as in the left precentral gyrus compared to controls. Direct comparison of the two patient groups revealed more atrophy in lvPPA for left temporal lobe and temporo-parietal areas. The reverse contrast showed more atrophy for the nfvPPA group in the right middle and inferior frontal gyri, superior frontal gyrus and bilateral supplementary motor area. These findings are consistent with previous studies (see above and Table 1).

**Figure 5:**
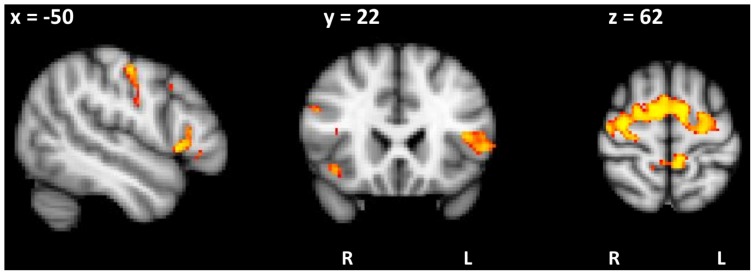
Brain regions in which grey matter intensity correlates significantly with a composite measure of relative vowel duration in words. A composite measure of pairwise variability for vowel duration in weak-strong and strong-weak polysyllable words was generated for each participant using weights from the discriminant function Model 2. Voxel-based morphometry analyses using diagnostic category (pooled PPA cohort and healthy control group) as a nuisance variable show that the index significantly covaried with atrophy in precentral gyrus, supplementary motor area and inferior frontal gyrus bilaterally. Bilateral atrophy in these areas was detected in the nonfluent variant of primary progressive aphasia but not the logopenic variant. Coloured voxels show regions that were significant in the analysis at *P*<0.05 FDR corrected. Clusters are overlaid on the MNI standard brain and reported at *t*>2.41.

Covariate analysis using the Silence+Duration Index from Model 1, considering only clusters >100 voxels, showed a significant relationship with atrophy in bilateral middle frontal gyrus and postcentral gyrus, in left precentral gyrus and supplementary motor area, and in right inferior frontal gyrus (pars opercularis) and insula cortex (see Table 5 and Figure S2). A second covariate analysis using the Duration Index from Model 2 (see Table 5 and Figure 5) showed a significant relationship with atrophy in supplementary motor area, precentral gyrus, and inferior frontal gyrus bilaterally.

**Table 5:** Clusters (>100 voxels) correlating with the Silence+Duration and Duration Indices generated from discriminant function analysis Models 1 and 2, respectively, including control participants and Primary Progressive Aphasia participants with MRI scans.

Contrast	Cluster Size	*T*-score	MNI coordinates of MAX (vox)			Regions in Cluster (Maxima location)
			X	Y	Z	
*Model 1* ^1^ *: Silence+Duration Index (variability of silence duration, proportion silence time, and vowel duration for weak-strong words)*						
Negative	4725	>1.67	69	54	61	Left precentral gyrus and supplementary motor area
	445	>1.67	50	45	67	Left postcentral gyrus
	441	>1.67	28	68	54	Right middle frontal gyrus/Inferior frontal gyrus (pars opercularis)
	276	>1.67	12	64	50	Right postcentral gyrus
	228	>1.67	65	68	56	Left middle frontal gyrus
	163	>1.67	25	53	34	Right insular cortex
	113	>1.67	32	73	28	Right orbito-frontal cortex/insula
	104	>1.67	72	63	45	Left precentral gyrus
	101	>1.67	18	73	50	Right inferior frontal gyrus (pars opercularis)
*Model 2* ^2^ *: Duration Index (relative vowel duration for weak-strong and strong-weak words)*						
Negative	3759	>2.41	52	20	28	Right inferior frontal gyrus/bilateral supplementary motor area
	345	>2.41	–64	4	34	Left precentral gyrus
	325	>2.41	–8	–36	64	Bilateral precentral gyrus
	259	>2.41	–52	18	2	Left inferior frontal gyrus
	200	>2.41	42	26	2	Right inferior frontal gyrus

**Note**. *P*<0.05 FDR corrected; ^1^ see Figure S2; ^2^ see Figure 4.

## Discussion

The aim of the study was to determine whether a set of instrumental measures of speech production distinguished cases of nonfluent variant primary progressive aphasia (nfvPPA), and more specifically cases of nfvPPA with AOS, from those with the logopenic variant (lvPPA) and healthy controls. If successful, use of these measures could shift the field from its reliance on diagnosis by expert perceptual judgment to use of more objective markers of speech. Hypotheses were largely supported, with several measures of silences during reading and of relative vowel duration within polysyllabic words differentiating the nfvPPA cases from lvPPA and control participants. Discriminant function analyses revealed that a simple measure of relative vowel duration from a polysyllabic word repetition test best differentiated the nfvPPA cases, showed high agreement with expert judgment on presence of AOS in nfvPPA cases (88%), and covaried significantly with grey matter intensity in regions of interest within the speech motor network. While amount and variability of silent periods during reading also discriminated the PPA and control groups, silence measures did not significantly increase the predictive accuracy of a model that included the relative duration measure.

The most informative acoustic measure was the pairwise variability index of vowel duration for the first two syllables of 3-syllable strong-weak words (PVI_Duration_SW) (e.g. DI-no-saur) and for weak-strong words (PVI_Duration_WS) (e.g. po-TA-to). This measure captures the degree of contrastiveness across two adjacent syllables in a word, with listeners typically rating words with lower values as equally stressed and robotic-sounding [25]. The findings are consistent with hypotheses that individuals with AOS have difficulty planning or programming multi-syllabic strings [47], [48]. Given that the measure of intensity contrastiveness did not differentiate the control and patient participants in this task, the deficit in the nfvPPA patients with AOS appears to be related specifically to controlling the durational relations across syllables within multisyllabic units of speech, rather than controlling intonation in production of lexical stress (i.e. intensity and fundamental frequency contours). Findings suggest that individuals with PPA need to be impaired in control of relative vowel duration across both types of polysyllabic words (strong-weak and weak-strong patterns) to be confidently classified as nfvPPA with AOS by the model presented here and to be perceived by expert clinicians as having the feature of equal stress characteristic of AOS.

While the measure of relative vowel duration was able to successfully categorise 84% of the study sample, some misclassifications occurred. The majority of these can be explained by those cases of nfvPPA who were judged clinically to have no AOS (accounting for four of the five misclassified nfvPPA cases). Only one misclassification occurred where lvPPA was categorized as nfvPPA (case 6) and the reason for this is less clear. The individual was a native Dutch speaker, but accented English did not appear to be the cause of her misclassification, given another native Dutch speaker was misclassified as lvPPA by the model. She had been diagnosed with PPA in the Frontotemporal Dementia Research Group clinic two years prior to the study but, on family report, had the longest history of symptoms in the sample, at 12 years, and was one of the more severely impaired in the lvPPA group (although severity cannot fully explain this result as other severe cases in the lvPPA group were correctly classified by the model). Unfortunately, no Pittsburgh compound B scan was available to confirm the lvPPA diagnosis. Despite these exceptions, overall the results clearly indicate the utility of these acoustic measures of speech production for the majority of cases.

In further support of these acoustic measures, composite indices of silence and/or relative vowel duration behaviors during speech generated from the discriminant function analyses were significantly correlated with reduced grey matter intensity in regions of interest within the speech motor control network. Higher Model 1 index values (i.e. more silence time during reading, lower contrastiveness of vowel duration in polysyllabic words with weak-strong stress) were associated with reduced grey matter intensity bilaterally in middle frontal gyrus and postcentral gyrus; and unilaterally in left precentral gyrus and supplementary motor area and in right inferior frontal gyrus (pars opercularis) and insula cortex. Higher Model 2 values (i.e. lower contrastiveness of vowel duration in both weak-strong and strong-weak stressed words) were associated with reduced grey matter intensity bilaterally in precentral gyrus, supplementary motor area, and inferior frontal brain regions. As observed here, AOS is strongly associated with lesions in the premotor cortex and/or inferior frontal gyrus (pars opercularis) [29] [30]. These regions are considered centrally involved in development and refinement of speech motor programs specifying temporally and spatially coordinated multi-articulator movements for syllable production [49], [50]. As well, supplementary motor area has been associated with preparation and initiation of speech sounds and syllables as well as reduced intonational and rhythmic variation in speech [50], [51], [52]. Regions specifically associated with the Model 1 index likely were related to increased silences during reading and included: bilateral middle frontal gyrus and postcentral gyrus and right insula cortex. These silences represent reductions in intensity of the speech signal and capture primarily pauses, hesitations, and prolonged transitions between speech sounds (e.g., stop gaps); all behaviours that would contribute to the percepts of nonfluent and segmented speech routinely used to describe AOS [1], [4]. The dorsolateral prefrontal cortex within middle frontal gyrus is associated with sustaining attention and working memory, consistent with proposals that the segmentation of AOS is due to a reduction in working memory buffer capacity, with only one syllable being uploaded and programmed at a time [47], [48]. Involvement of postcentral gyrus supports a sensori-motor integration impairment in AOS, critical to processing feedback and correcting and refining motor programs [49], [53]. The right insula appears to be involved in peripheral arousal, when emotional states or feelings about a stimulus are re-accessed and influence other processes including working memory and motor behaviors [54], [55]. This peripheral arousal underlies anticipation of endogenous stimuli and perceptual feedback, particularly for more difficult tasks [56]. Future work may reveal whether atrophy involving the right insula in these patients contributes to their working memory impairment or affects the anticipation of endogenous sensory feedback, further disrupting speech sensori-motor feedback loops.

Notably, the lvPPA group demonstrated atrophy extending from temporo-parieto-occipital junction through to posterior inferior frontal gyrus, but isolated to the left hemisphere. The nfvPPA group, on the other hand, demonstrated left frontal atrophy overlapping with the lvPPA group, but involvement of right homologue areas as well as bilateral supplementary motor area. This shared involvement of the left frontal regions may explain the diagnostic challenge in differentiating these two groups. However, the findings reported here suggest that specific apraxic speech features emerge or reach a critical threshold when cortical regions for speech motor control are affected bilaterally, preventing or reversing any cross-hemispheric compensatory mechanisms [57].

The discriminant function analysis allowed comparison of the two participant groups in this study sample (lvPPA and nfvPPA), with data from control participants provided in each figure for comparison. The generalisability of the resulting model to the three-group case, or to a different sample of patients, will be influenced by a range of variables. Given that the lvPPA and nfvPPA patients are already well differentiated from control speakers by virtue of their presentation in the neurologist’s office, we would argue that the diagnostic dilemma lies is in differentiating the two patient groups. Here, we have made considerable progress toward this goal, using measures that do not rely on expert judgment and that have potential for automation for ease of use. Groups were best differentiated by the PVI_Vowel Duration measure.

The measurement of temporal properties of speech in progressive AOS is not entirely novel. Laganaro *et al*. [58] measured durations of consonant-vowel syllables across a range of speaking tasks for French individuals with progressive AOS. They reported a significant effect of a syllable’s frequency in the French language on syllable duration, with less frequent syllables being more susceptible to abnormal lengthening. This effect was independent and more robust than other variables tested, including phoneme or lexical frequency, phonological neighborhood density, position in words, or frequency of adjacent syllables. Similar to prior hypotheses [59], Laganaro and colleagues argued that syllable lengthening in AOS follows a gradient of syllable frequency rather than being a categorical effect of high versus low frequency [60]. Here, syllable frequency was not systematically varied but most syllables were of high frequency. While the structure of lexical stress in French and English differ, with English being stress-timed and French syllable-timed, Courson *et al*. [27] have shown that the PVI measure as used here has potential for detecting disruptions in relative durations within word in French as well as English.

Data presented here, and by Laganaro *et al*. [58], support the view that a core deficit in AOS involves retrieving and implementing syllabic motor plans. Findings indicate that syllable or vowel lengthening is not a blanket strategy applied to slow down speech and allow more time for an impaired motor planning mechanism to achieve articulatory planning goals, but rather is sensitive to specific variables such as syllable frequency and syllable and word structure. Studies of AOS after stroke have reported a similar effect of syllable frequency on likelihood of phoneme distortion and substitution errors [61], [62], [63]. These factors provide insight into the underlying cause of the symptoms in AOS and can guide more targeted approaches in emerging behavioral interventions.

While the measurements of silences during reading did not contribute significantly to the statistical model, silence duration and variability of silence duration were significant univariate predictors of diagnostic group. Here, silence was calculated objectively based on variations in the intensity contour across the reading sample [26], [39]. Excessively longer or more frequent silences can be indicative of either retrieval or motor planning/programming disruptions. Here, both patient groups demonstrated similar levels of silence time within their reading sample, as a proportion of total sample length, and significantly greater silence time than control speakers. However, the nfvPPA cases showed significantly longer (median of 236 ms) and more variable silence durations than both lvPPA (median of 126 ms) or control (median of 83 ms) groups. Average speaking rate for healthy speakers is about 5 syllables per second, or 200 ms per syllable (Yorkston *et al*., 1999). This implies that the nfvPPA cases had fewer but longer silences, almost three times longer than normal, while the lvPPA cases had more silences but these were typically only 50% longer than normal. These findings are likely task-dependent; the reading task may have facilitated lexical retrieval for the lvPPA group (or both groups) such that long word-finding pauses were not observed. It is possible that lengthened silence durations may emerge for the lvPPA group in a picture description or narrative task, where word-retrieval demands are greater. The abnormal silence durations of the nfvPPA group, in the context of a reading task with low demands on lexical retrieval, support a post-lexical phonetic-motoric basis to the impairment for this group.

Non-native English speakers were included in the study and this decision warrants some discussion. It is customary to restrict patient samples in research studies to those whose native language is the same as the researchers, to avoid assumptions about language competence. This is less critical in studies of motor speech disorders where the focus is on integrity of the motor system. However, a non-native accent can influence some measures of motor speech production. The relative vowel duration measures for words of different stress patterns are susceptible given that the world’s languages fall roughly into three categories – stress-timed (e.g. English, German), syllable-timed (e.g. French, Singaporean English), and mora-timed (e.g Japanese) [64]. Here, eight of the 41 PPA participants were non-native English speakers, although fluent in English pre-onset of PPA. Of these eight, the native language was in the stress-timed group for six participants and so should pattern with English, with low values on the PVI measure indicating abnormality. The other two, Latvian and Malayalam, are both syllable-timed languages. Latvian has a natural tendency to equal vowel durations within word [65], but Malayalam has some features of stress-timed languages such as reduced vowel duration in unstressed syllables [66], as does English, that would give rise to larger PVI values. While it is possible that these two cases were falsely identified as nfvPPA based on their non-native accent, the nfvPPA diagnosis is supported in both cases by negative Pittsburgh compound B findings. Furthermore, recomputing our analyses, with the non-native English speakers excluded, did not alter the major findings or conclusions of the study (see Tables S1 and S2).

On a related issue, several of the words selected as stimuli contained the post-vocalic/pre-consonantal rhotic (‘r’) sound in the weak syllable (e.g. the­rmometer, motorbike). As noted above, this is silent in Australian English but is expressed in American English, for example. For rhotacized vowels, these weak vowel durations will be lengthened resulting in a smaller magnitude PVI value. In this study, the stimulus words were selected retrospectively from existing recordings of responses to the SYDBAT word repetition test [32]. Future studies should restrict stimuli to contain non-rhoticized weak vowels for direct comparison to the results reported here.

The findings clearly require testing with a larger sample and independent replication. As automatic speech recognition methods develop [67], it may become more feasible to include an acoustic measure(s) of phoneme distortion to identify those cases judged to have AOS without equalized stress over words or phrases [4]. In addition, the acoustic measures used here in future may be combined with measures of grammatical structure in language production to aid in differentiating cases of nfvPPA with predominant AOS versus predominant agrammatism.

Pittsburgh compound B scanning is not routinely available to guide diagnosis and, here, was not available for all cases. The model’s accuracy was improved when the three nfvPPA cases returning positive Pittsburgh compound B results were excluded from the final discriminant function analysis. Of interest, when the PVI data from these cases were entered into this model formula, they were classified as lvPPA, consistent with Pittsburgh compound B test results. While a number of participants did not undergo this test, the behavioural findings correlated well with findings from group analyses of structural MRI data, lending support to their robustness.

While acoustic analysis of speech has not yet become routine in clinical practice, this has been a goal for some decades [68]. The measures used here are taken from commonly used assessment tasks. They require high quality audio recordings and these are now easily made using smartphone applications, such as Pocket Wavepad (NCH Software) for speech sample acquisition and Praat speech analysis software; both are freeware. Automated measurement and analysis tools are already available or are being developed for all measures reported. This will aid development of standardized and rapidly implemented assessments that reduce reliance on expert judgment in both research and clinical contexts. At this time, these measures must be extracted manually, limiting clinical feasibility. However, the relative vowel duration measure is highly correlated with perceptual judgments of “goodness” of lexical stress. For the present, perceptual judgment of lexical stress in production of these three-syllable words, with attention to relative duration of vowels in syllables, may suffice. In particular, data presented here suggest that prolongation of the very brief initial syllables in weak-strong words justifies suspicion of AOS.

In summary, we show that a simple measure of relative vowel duration from a word repetition task can provide a useful diagnostic adjunct for detecting AOS in PPA, and thereby distinguishing between the majority of lvPPA and nfvPPA patients. The measure covaries as expected with well-known motor speech regions, such as precentral gyrus, supplementary motor area and inferior frontal gyrus bilaterally, which were only affected in the nfvPPA group. Both the relative vowel duration and silence measures diminish the need for expert judgment and provide quantitative metrics to measure change with behavioural intervention. Reliable diagnosis based on behavioural symptoms will also aid rapid and cost-effective identification of patients who may be suitable for future pharmacologic interventions.

## Supporting Information

Figure S1 **Differences in grey matter intensity between healthy adults and individuals with logopenic or nonfluent variants of primary progressive aphasia.** Voxel-based morphometry analyses showing brain areas of decreased grey matter intensity in a) lvPPA vs. Controls (red-yellow); b) nfvPPA vs. Controls (red-yellow); c) lvPPA vs. nfvPPA (green); and d) nfvPPA vs. lvPPA (blue). Coloured voxels show regions that were significant in the analysis at *P*<0.05 FDR corrected. Clusters are overlaid on the MNI standard brain and reported at *t*>3.27.
(TIFF)

Figure S2 **Brain regions in which grey matter intensity correlates significantly with a composite measure of silences during reading and relative vowel duration in words.** A composite measure of variability of silence duration, percent silence time, and vowel duration for weak-strong words was generated for each participant using weights from the discriminant function Model 1. Voxel-based morphometry analyses show that the index significantly covaried with atrophy in right precentral gyrus and right postcentral gyrus, affected in the nonfluent variant of primary progressive aphasia but not the logopenic variant. Only cluster sizes greater than 100 voxels were considered. Bilateral atrophy in these areas was detected in the nonfluent variant of primary progressive aphasia but not the logopenic variant. Coloured voxels show regions that were significant in the analysis at *P*<0.001 FDR uncorrected; no regions reached significance at *P*<0.05 FDR corrected. Clusters are overlaid on the MNI standard brain and reported at *t*>3.27.
(TIFF)

Table S1 **Comparison between healthy controls (N  =  17) and individuals with logopenic (lvPPA, N  =  20) or nonfluent variant (nfvPPA, N  =  14) Primary Progressive Aphasia on acoustic measures of speech (total N  =  51), with 1 lvPPA and 7 nfvPPA cases with non-native English background excluded from the original 58 participant sample.** Bold font indicates where a comparison changed from significant with the 58-participant sample to non-significant with the 51-participant sample. Note, that the PVI_Duration measures survive as variables that significantly differentiate the lvPPA and nfvPPA groups, while no silence measures survive.
(DOCX)

Table S2 **Results of multivariate discriminant function analyses with aphasia variant as the dependent variable, with non-native English speakers included (Models 1 and 2) or excluded (Model 3).** Note, that Models 1 and 2 are identical to Table 4 in the main text (see note below for details).
(DOCX)

Method S1 **Description of method used for measuring silences in connected speech.**
(DOCX)
