# Correction: Logopenic and Nonfluent Variants of Primary Progressive Aphasia Are Differentiated by Acoustic Measures of Speech Production

**DOI:** 10.1371/journal.pone.0121941

**Published:** 2015-03-26

**Authors:** The PLOS ONE Staff

The following information is missing from the Funding section.

National Health and Medical Research Council (NHMRC) of Australia Project Grant (No. 632763, in part, to KJB).
