# Supplementary figures and images for: Logopenic and Nonfluent Variants of Primary Progressive Aphasia Are Differentiated by Acoustic Measures of Speech Production
Source: PLoS One. 2014 Feb 28;9(2):e89864. doi: 10.1371/journal.pone.0089864 (PMC3938536; doi:10.1371/journal.pone.0089864)

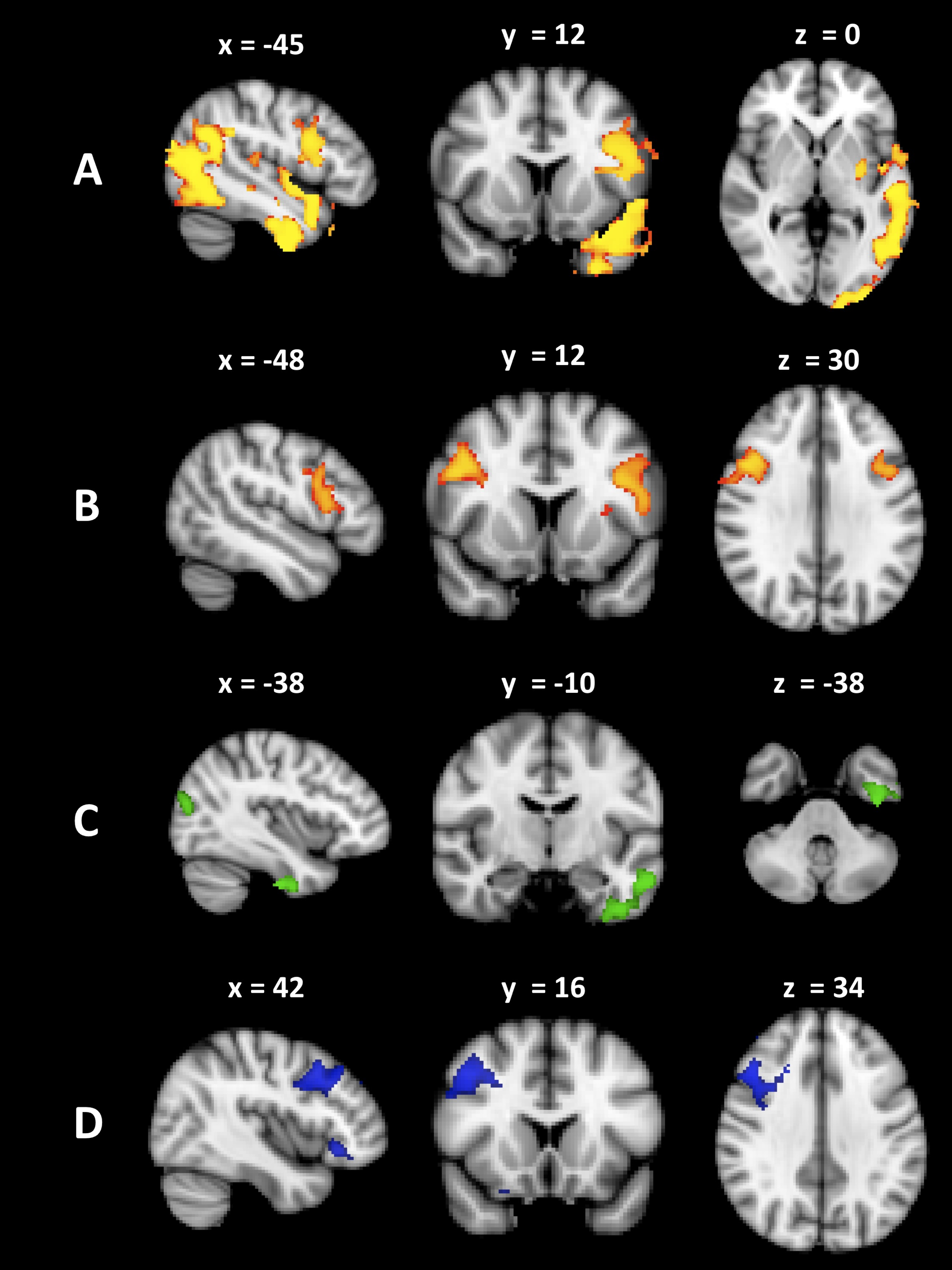

Supplement: Figure S1 — Differences in grey matter intensity between healthy adults and individuals with logopenic or nonfluent variants of primary progressive aphasia. Voxel-based morphometry analyses showing brain areas of decreased grey matter intensity in a) lvPPA vs. Controls (red-yellow); b) nfvPPA vs. Controls (red-yellow); c) lvPPA vs. nfvPPA (green); and d) nfvPPA vs. lvPPA (blue). Coloured voxels show regions that were significant in the analysis at P<0.05 FDR corrected. Clusters are overlaid on the MNI standard brain and reported at t>3.27. (TIFF) [file pone.0089864.s001.tiff]

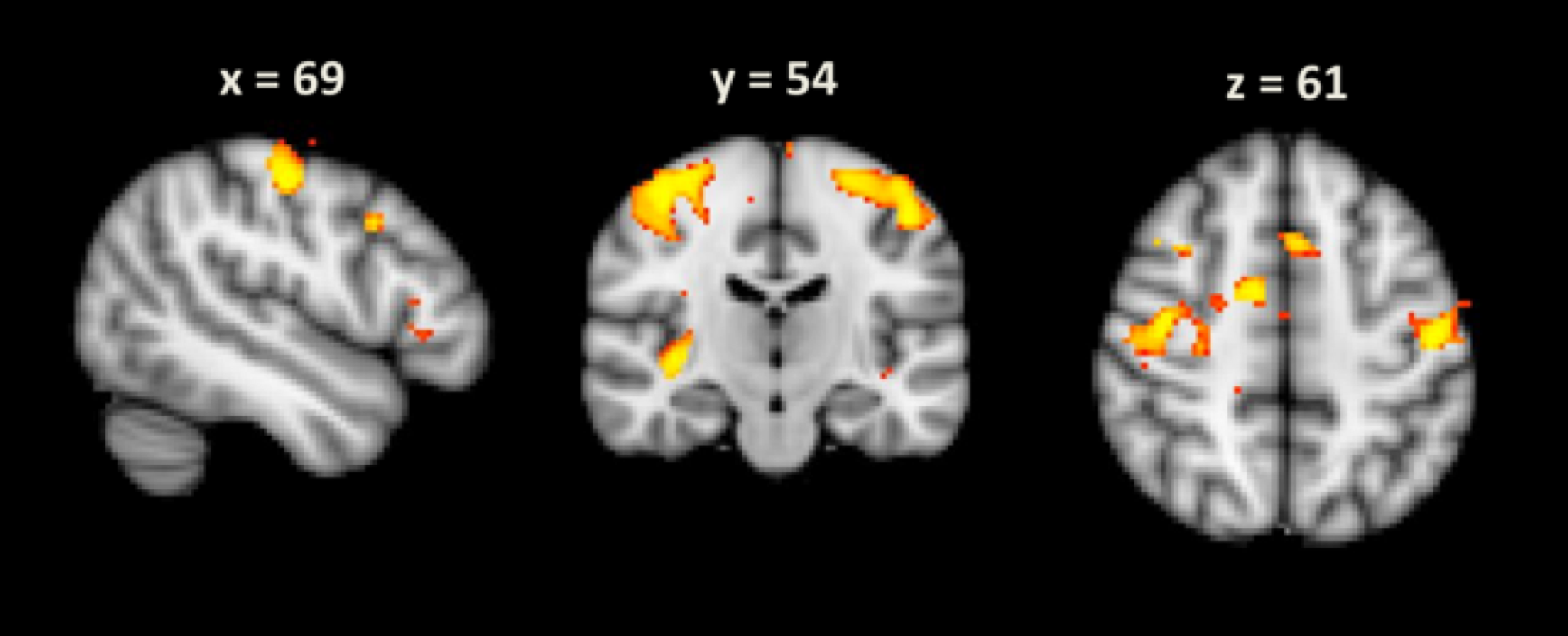

Supplement: Figure S2 — Brain regions in which grey matter intensity correlates significantly with a composite measure of silences during reading and relative vowel duration in words. A composite measure of variability of silence duration, percent silence time, and vowel duration for weak-strong words was generated for each participant using weights from the discriminant function Model 1. Voxel-based morphometry analyses show that the index significantly covaried with atrophy in right precentral gyrus and right postcentral gyrus, affected in the nonfluent variant of primary progressive aphasia but not the logopenic variant. Only cluster sizes greater than 100 voxels were considered. Bilateral atrophy in these areas was detected in the nonfluent variant of primary progressive aphasia but not the logopenic variant. Coloured voxels show regions that were significant in the analysis at P<0.001 FDR uncorrected; no regions reached significance at P<0.05 FDR corrected. Clusters are overlaid on the MNI standard brain and reported at t>3.27. (TIFF) [file pone.0089864.s002.tiff]
