# Supplementary material for: Logopenic and Nonfluent Variants of Primary Progressive Aphasia Are Differentiated by Acoustic Measures of Speech Production
Source: PLoS One. 2014 Feb 28;9(2):e89864. doi: 10.1371/journal.pone.0089864 (PMC3938536; doi:10.1371/journal.pone.0089864)
Supplement: Table S1 — Comparison between healthy controls (N = 17) and individuals with logopenic (lvPPA, N = 20) or nonfluent variant (nfvPPA, N = 14) Primary Progressive Aphasia on acoustic measures of speech (total N = 51), with 1 lvPPA and 7 nfvPPA cases with non-native English background excluded from the original 58 participant sample. Bold font indicates where a comparison changed from significant with the 58-participant sample to non-significant with the 51-participant sample. Note, that the PVI_Duration measures survive as variables that significantly differentiate the lvPPA and nfvPPA groups, while no silence measures survive. (DOCX) [file pone.0089864.s003.docx]

**Table S1**. Comparison between healthy controls (N = 17) and individuals with logopenic (lvPPA, N = 20) or nonfluent variant (nfvPPA, N = 14) Primary Progressive Aphasia on acoustic measures of speech (total N = 51), with 1 lvPPA and 7 nfvPPA cases with non-native English background excluded from the original 58 participant sample. Bold font indicates where a comparison changed from significant with the 58-participant sample to non-significant with the 51-participant sample. Note, that the PVI_Duration measures survive as variables that significantly differentiate the lvPPA and nfvPPA groups, while no silence measures survive.

| **Measure** | **Omnibus test^1^** | **Group** | **Mean (SD)** | **Post-hoc tests** |
| --- | --- | --- | --- | --- |
| Proportion Silence Time ^1^ | *F*(2,47) = 4.516, *P* = 0.016 | lvPPA | 0.59 (0.12) | lvPPA - nfvPPA ^ns^ |
|  |  | nfvPPA | 0.57 (0.15) | lvPPA – Control * |
|  |  | Control | 0.48 (0.09) | **nfvPPA-Control ^ns^** |
| Median Silence Duration (ms) | *F*(2,47) = 10.495, *P* = 0.000 | lvPPA | 117.6 (33.5) | **lvPPA-nfvPPA ^ns^** |
|  |  | nfvPPA | 147.0 (58.6) | lvPPA-Control * |
|  |  | Control | 83.3 (27.2) | nfvPPA-Control *** |
| Variability of Silence Duration | *F*(2,47) = 8.894, *P* = 0.000 | lvPPA | 78.5 (31.1) | **lvPPA-nfvPPA ^ns^** |
|  |  | nfvPPA | 102.1 (50.1) | lvPPA-Control * |
|  |  | Control | 49.7 (27.6) | nfvPPA-Control *** |
| Median PVI_Duration_WS | *F*(2,47) = 5.817, *P* = 0.006 | lvPPA | 118.4 (15.5) | lvPPA-nfvPPA ** |
|  |  | nfvPPA | 92.2 (31.2) | lvPPA-Control ^ns^ |
|  |  | Control | 109.7 (18.9) | **nfvPPA-Control ^ns^** |
| Median PVI_Duration_SW | *F*(2,47) = 6.515, *P* = 0.003 | lvPPA | 91.7 (14.6) | lvPPA-nfvPPA ** |
|  |  | nfvPPA | 66.0 (29.9) | lvPPA-Control ^ns^ |
|  |  | Control | 80.7 (16.0) | **nfvPPA-Control ^ns^** |
| Median PVI_Intensity_WS | *F*(2,47) = 2.169, *P* = 0.126 |  |  |  |
| Median PVI_Intensity_SW | *F*(2,47) = 0.066, *P* = 0.936 |  |  |  |

^1^ Proportion of silence arcsin transformed, Median Silence Duration and Variability of Silence Duration square root transformed, means represent nontransformed values; ns = nonsignificant, **P* < 0.05, ** *P* < 0.01, *** *P* < 0.001. PVI = Pairwise Variability Index, WS = weak-strong words (e.g. potato), SW = strong-weak words (e.g. dinosaur).
