# Supplementary material for: Logopenic and Nonfluent Variants of Primary Progressive Aphasia Are Differentiated by Acoustic Measures of Speech Production
Source: PLoS One. 2014 Feb 28;9(2):e89864. doi: 10.1371/journal.pone.0089864 (PMC3938536; doi:10.1371/journal.pone.0089864)
Supplement: Table S2 — Results of multivariate discriminant function analyses with aphasia variant as the dependent variable, with non-native English speakers included (Models 1 and 2) or excluded (Model 3). Note, that Models 1 and 2 are identical to Table 4 in the main text (see note below for details). (DOCX) [file pone.0089864.s004.docx]

**Table S2**. Results of multivariate discriminant function analyses with aphasia variant as the dependent variable, with non-native English speakers included (Models 1 and 2) or excluded (Model 3). Note, that Models 1 and 2 are identical to Table 4 in the main text (see note below for details).

|  |  | **Unstandardized Coefficient** | | **Standardized Coefficient** |  |  |
| --- | --- | --- | --- | --- | --- | --- |
| **Predictor Variables** | ***r*^2^** | **B** | **Standard Error** | **Beta** | ***t*** | ***P*** |
| **Model 1** |  |  |  |  |  |  |
| *F*(3,37) = 10.541, *P* = 0.000 | 0.461 |  |  |  |  |  |
| (Constant) |  | 2.387 | 0.460 |  | 5.192 | 0.000 |
| Proportion of silence time ^1^ |  | -2.057 | 0.638 | -0.611 | -3.224 | 0.003 |
| Variability of silence duration |  | 2.991 | 0.987 | 0.591 | 3.030 | 0.004 |
| Median PVI_Duration_WS |  | 0.010 | 0.003 | -0.520 | -3.785 | 0.001 |
|  |  |  |  |  |  |  |
| **Model 2** |  |  |  |  |  |  |
| *F*(2,35) = 15.471, *P* = 0.000 | 0.471 |  |  |  |  |  |
| (Constant) |  | 1.763 | 0.247 |  | 7.129 | 0.000 |
| Median PVI_Duration_WS |  | -0.008 | 0.003 | -0.422 | -2.771 | 0.009 |
| Median PVI_Duration_SW |  | -0.007 | 0.003 | -0.347 | -2.278 | 0.029 |
|  |  |  |  |  |  |  |
| **Model 3** ^2^ |  |  |  |  |  |  |
| F(2,31) = 7.823, P = 0.002 | 0.343 |  |  |  |  |  |
| (Constant) |  | 1.637 | .330 |  | 4.959 | 0.000 |
| Median PVI_Duration_WS |  | -0.006 | 0.003 | -0.328 | -1.929 | 0.063 |
| Median PVI_Duration_SW |  | -0.007 | 0.003 | -0.350 | -2.061 | 0.048 |

Model 1 includes all participants (N = 41); Model 2 excludes three nfvPPA patients with contradictory findings on Pittsburgh compound B scanning (N = 38); Model 3 excludes 1 lvPPA and 7 nfvPPA cases with non-native English background (N = 33); ^1^ arcsin transformed; ^2^ Selection of variables for Model 3 is described in Method of the main text (refer to Table S1 for precursor univariate analyses).
