# Supplementary material for: Logopenic and Nonfluent Variants of Primary Progressive Aphasia Are Differentiated by Acoustic Measures of Speech Production
Source: PLoS One. 2014 Feb 28;9(2):e89864. doi: 10.1371/journal.pone.0089864 (PMC3938536; doi:10.1371/journal.pone.0089864)
Supplement: Method S1 — Description of method used for measuring silences in connected speech. (DOCX) [file pone.0089864.s005.docx]

**Method used for measuring silences in connected speech**

Each speech sample was segmented and analyzed using a freely available acoustic analysis software program, Praat [[1](#_ENREF_1)]. Speech samples were segmented and analyzed using automated scripts designed to derive information from large batches of data containing multiple samples. Silences were identified from the intensity contour using a modified version of the techniques described by [[2](#_ENREF_2),[3](#_ENREF_3)]. Silences were identified from the intensity contour using three thresholds: (a) *intensity threshold*, (b) *minimum silence duration* (15 ms), and (c) *minimum speech duration* (30 ms). Silence segments were defined as the parts of the intensity contour that fell below the *intensity threshold*. Silence sections that were shorter than 15 ms were classed as speech and concatenated with the adjacent speech sections. Speech sections that were shorter than 30 ms were classed as silences and concatenated with the adjacent silences. The *intensity threshold* was set to 0.65 of the distance between the reference intensity (equal to 0.95 of the maximum intensity) and floor intensity (minimum). Reference intensity selection of 0.95 of the maximum intensity has been found more robust than use of the maximum, median, or modal intensities due to irregular bursts of energy that often occur with sporadically loud syllables or short phrases in spontaneous speech (e.g., emphatic stress). Visual inspection of the spectrum has shown that 0.95 of the maximum intensity represents the typical intensity of syllable peaks, whereas maximum intensity reflects a single observation interval and is less reliable than use of the reference intensity threshold described. The timing measures derived from this method included proportion of silence time (summed duration of silences / total duration of sample), median duration of silences, and variability of silence duration (median absolute deviation of silence duration). These measures have demonstrated reliability, stability and sensitivity to change and impairment in both healthy and pathological groups [[4-8](#_ENREF_4)].

1. Boersma P (2001) Praat, a system for doing phonetics by computer. Glot International 5: 341-345.

2. Green JR, Beukelman DR, Ball LJ (2004) Algorithmic estimation of pauses in extended speech samples of dysarthric and typical speech. J Med Speech Lang Pathol 12: 149-154.

3. Rosen KM, Murdoch BE, Folker JE, Vogel AP, Cahill L, et al. (2010) Automatic method of pause measurement for normal and dysarthric speech. Clin Linguist Phon 24: 141-154.

4. Mundt JC, Vogel AP, Feltner DE, Lenderking WR (2012) Vocal Acoustic Biomarkers of Depression Severity and Treatment Response. Biol Psychiatry 72: 580-587.

5. Vogel AP, Fletcher J, Maruff P (2010) Acoustic analysis of the effect of sustained wakefulness on speech. J Acoust Soc Am 128: 3747-3756.

6. Vogel AP, Fletcher J, Snyder PJ, Fredrickson A, Maruff P (2011) Reliability, stability, and sensitivity to change and impairment in acoustic measures of timing and frequency. J Voice 25: 137-149.

7. Vogel AP, Maruff P (2008) Comparison of voice acquisition methodologies in speech research. Behav Res Methods 40: 982–987

8. Vogel AP, Shirbin C, Churchyard AJ, Stout JC (2012) Speech acoustic markers of early stage and prodromal Huntington's disease: A marker of disease onset? Neuropsychologia 50: 3273-3278.
